# Mental realities—the concept of mental disorder and the mind-body problem

**DOI:** 10.3389/fpsyg.2013.00809

**Published:** 2013-11-05

**Authors:** Michael Jungert

**Affiliations:** German National Academic Foundation, Bonn, Germany

**Keywords:** concept of mental disorder, reductionism, mind-body problem, PTSD, trauma

From a philosophical point of view, the subject of Thomas Schramme's article seems to be well-known as he addresses one of the most prominent debates in both classical and contemporary philosophy: the mind-body problem. Of course, Schramme does not attempt to broadly cover this general issue. He rather focuses on the neglect of philosophical approaches within the context of the search for a sound definition of mental disorders that led to conceptual as well as theoretical problems for psychology and psychiatry. As a result of this neglect, Schramme argues, psychiatry is facing a make-believe dilemma that implies either Cartesian dualism or reductionism/eliminativism when trying to save the notion of “mental disorder.” In a nutshell, this apparent dilemma for current psychiatry goes as follows: Either we try to save the notion of mental disorder by claiming an independent sphere of the mental and end up with the implausibility of substance dualism. Or we attempt to avoid this problem by means of consistent somatization and a naturalistic reduction of mental terms and phenomena, thereby in fact disposing of any substantial meaning of *mental* disorder. Hence, psychiatry seems to be stuck “between the Scylla of reduction and the Charybdis of dualism” (Schramme, 2013, p. 2).

As Schramme convincingly shows, the prevailing acceptance of this alleged dilemma in psychiatry is due to some fundamental misconceptions and the “limited awareness of the philosophical debate on the mind-body problem” (Schramme, 2013, p. 1). He demonstrates this claim by discussing two prominent positions in the philosophy of mind which—albeit in quite different ways—eliminate the level of psychological explanation and at the same time any significant meaning of mental disorder: identity theory and eliminative materialism. While his discussion necessarily remains cursory, it covers the most important objections against both theories. For philosophers, the most surprising aspect in Schramme's analysis of this rather well-known controversy consists of the fact that psychiatry has so far to a large extent ignored important conceptual differentiations that could help to avoid false conclusions like the idea that the concept of mental disorder compellingly implies “a Cartesian view of the mind-body problem, that minds and brains are separable and entirely distinct realms, an approach that is inconsistent with modern philosophical and neuroscientific views” (Stein et al., 2010, p. 1760). A closer look at those “modern philosophical views” would have shown that there is no necessary connection between “mental” and substance dualism but rather different (e.g., phenomenological and narrative) approaches that try to define and describe a rich concept of mental illness without falling back to Cartesianism (for an overview see Perring, 2010).

Schramme succeeds in demonstrating the general problems of reductive and eliminative theories and shows that both types of theories do not provide compelling reasons for rejecting “the possibility of an independent conceptualization of mental illness” (Schramme, 2013, p. 3). Only to a lesser extent, however, does he address the specific features of mental illness that determine its conceptual autonomy and immunize it against scientific naturalism and reductive explanation. As an extension to Schramme's line of argument, I will therefore briefly discuss the case of *posttraumatic stress disorder* (PTSD).

PTSD has become well-known in the context of war veterans who were—passively or actively—involved in extreme forms of physical or psychological violence. After having returned from mission, sometimes years or even decades later, some of them start to re-experience certain episodes, for instance the unintended killing of civilians. These episodes appear as very lively, uncontrollable autobiographical memories that emotionally affect the patient and inevitably arrest his attention. By forcing the patient to relive the traumatic experience again and again, such memories create “black holes” (Pitman and Orr, 1990, p. 469) in the narrative reality of the person, unintentionally attracting his attention without being able to successfully integrate the remembered event into his life story. While being an inerasable part of the *historic reality* of the person, it cannot at the same time be accepted as truly belonging to oneself and therefore cannot be integrated into the persons' *narrative reality* (Jungert, 2013, p. 202). Thus, there remains a *foreign body* in the life story of the person that constantly causes flashbacks and induces the persons' suffering from his past (Hampe, 2007, p. 92).

Why is PTSD a good example for the irreducibility of psychological explanation that Schramme seeks to defend? Most notably, because it reveals the fundamental problems that result from any attempt to reduce the internal perspective of human minds to the external perspective on human brains. As described above, traumatic disorder can be understood as a break into a persons' history caused by the traumatic event. The awareness of this break is tied to the categories of meaning and subjective reality—the idea of something being true *for* someone—which again can only be captured if one assumes some kind of mental reality and the existence of an internal perspective. It is *by reconstructing these internal perspectives* that psychiatry is able to get access to traumatic disorders and—at least in some cases—to find a way of dealing with them conjointly with the patient.

As a matter of principle, even the most sophisticated neuroscience or biological psychiatry would not be able to approach mental disorders like PTSD appropriately, because the recognition or analysis of internal perspectives is not part of their methodological repertoire, nor can it be grasped by its basic concepts. Instead, by trying to describe psychological phenomena exclusively by using somatic terms and categories, they in fact eliminate those perspectives, because “nothing is true for somatic structures on their own, i.e., they cannot be treated as something with an internal perspective” (Hampe, 2007, p. 100). Saving the sphere of internal perspective, however, does not necessarily imply to invoke substance dualism. In consonance with Schramme's reasoning, it is enough to consider the mental and the somatic dimension as phenomenologically different, but complementary aspects of one substance.
